# Mobile Phone Syndromic Surveillance for Respiratory Conditions in an Emergency (COVID-19) Context in Colombia: Representative Survey Design

**DOI:** 10.2196/50184

**Published:** 2024-10-17

**Authors:** Andres I Vecino-Ortiz, Deivis Nicolas Guzman-Tordecilla, Vidhi Maniar, Sandra Agudelo-Londoño, Oscar Franco-Suarez, Nathaly Aya Pastrana, Mariana Rodríguez-Patarroyo, Marino Mejía-Rocha, Jaime Cardona, Mariangela Chavez Chamorro, Dustin Gibson

**Affiliations:** 1 Health Systems Program International Health Department Johns Hopkins Bloomberg School of Public Health Baltimore, MD United States; 2 Pontificia Universidad Javeriana Bogota Colombia; 3 IMEK Cali Colombia; 4 Inter-American Development Bank Bogota Colombia

**Keywords:** mobile phone surveys, syndromic surveillance, COVID-19, public health surveillance, IVR, interactive voice response, survey, surveys, voice response, syndromic, surveillance, respiratory, pandemic, SARS-CoV-2, feasibility, data collection, public health, emergency, outbreak, mobile phone

## Abstract

**Background:**

Syndromic surveillance for respiratory infections such as COVID-19 is a crucial part of the public health surveillance toolkit as it allows decision makers to detect and prepare for new waves of the disease in advance. However, it is labor-intensive, costly, and increases exposure to survey personnel. This study assesses the feasibility of conducting a mobile phone–based respiratory syndromic surveillance program in a middle-income country during a public health emergency, providing data to support the inclusion of this method in the standard infection control protocols at the population level.

**Objective:**

This study aims to assess the feasibility of a national active syndromic surveillance system for COVID-19 disease in Colombia.

**Methods:**

In total, 2 pilots of syndromic mobile phone surveys (MPSs) were deployed using interactive voice response technology in Colombia (367 complete surveys in March 2022 and 451 complete surveys in April and May 2022). Respondents aged 18 years and older were sampled using random digit dialing, and after obtaining consent, they were sent a 10-minute survey with modules on sociodemographic status, respiratory symptoms, past exposure to COVID-19 infection and vaccination status, preferences about COVID-19 vaccination, and information source for COVID-19. Pilot 1 used a nationally representative sample while pilot 2 used quota sampling to yield representative results at the regional level. In this work, we assessed the performance characteristics of the survey pilots and compared the demographic information collected with a nationally representative household survey.

**Results:**

For both pilots, contact rates were between 1% and 2%, while participation rates were above 80%. The results revealed that younger, female, and higher educated participants were more likely to participate in the syndromic survey. Survey rates as well as demographics, COVID-19 vaccination status, and prevalence of respiratory symptoms are reported for both pilots. We found that respondents of the MPSs are more likely to be younger and female.

**Conclusions:**

In a COVID-19 pandemic setting, using an interactive voice response MPS to conduct syndromic surveillance may be a transformational, low-risk, and feasible method to detect outbreaks. This evaluation expects to provide a path forward to the inclusion of MPSs as a traditional surveillance method.

## Introduction

Surveillance systems that provide real-time and accurate information are increasingly critical for decision-making in public health, as demonstrated by the COVID-19 pandemic [1]. Commonly used indicators for monitoring the COVID-19 pandemic were the number of laboratory-confirmed cases, the number of persons hospitalized or in intensive care, and the number of COVID-19 deaths [2,3]. However, these indicators are highly dependent on the health systems’ capacity, might fail to represent real-time conditions, are often mediated by other factors such as health care access, and are less likely to detect less severe cases [4]. These challenges become more important in low- and middle-income countries (LMICs) where traditional surveillance tools are scarce. For this reason, new strategies to complement traditional epidemiologic surveillance are needed [5]. The World Health Organization (WHO) has recommended enhancing traditional epidemiologic surveillance systems with other tools such as syndromic surveillance, where individuals can self-report symptoms related to infection [5], improving the timeliness and coverage, at lower costs [6-9].

Mobile phones have the potential to be an efficient tool to perform syndromic surveillance [1,4,10] because of their widespread use. For example, in Colombia, it has been estimated that there are 133 mobile telephone subscriptions for every 100 people [11]. In addition, it has been identified that surveys using mobile phone technology have lower costs than household surveys [9]. Finally, mobile phone syndromic surveillance is easy to deploy in emergency contexts where social distancing is required.

Mobile phone–based syndromic surveillance systems used during the COVID-19 pandemic have been mainly passive and limited to mobile phone apps that require smartphone technology linked to contact-tracing apps [1,4,10,12-14], which could be limited in LMIC where the number of smartphones with internet access is limited [10]. Other studies in LMIC have used the interactive voice response (IVR) as an active surveillance system on behavior, exposure, knowledge, and perception related to COVID-19, but not as a syndromic surveillance system [6,15,16]. To our knowledge, there have been no published experiences of an active syndromic surveillance system on COVID-19 disease or other health emergencies using IVR as a data collection tool. We hypothesize that syndromic surveillance is a feasible option to conduct syndromic surveillance in respiratory emergencies. This study aims to assess the feasibility of a national active syndromic surveillance system for COVID-19 disease in Colombia.

## Methods

### Data and Surveillance Instrument

IVR surveys were developed and cognitive testing was performed [17]. Participant phone numbers were obtained through random digit dialing [18], which included a prefix ranging from 300 to 323 (which are the prefixes for all mobile phone numbers used in Colombia), followed by 7 digits randomly selected. Random digit dialing is a technique used in previous research to yield a random sample of phone numbers when a previous database does not exist or cannot be accessed for privacy reasons [19]. Respondents who were aged ≥18 years and provided consent, were considered eligible and could participate in a 10-minute survey that comprised the following topical modules: sociodemographic status, respiratory symptoms, previous COVID-19 infection and vaccination status, preferences about COVID-19 vaccination and information on COVID-19. On survey completion, respondents were given an airtime incentive of COL $4000 (around US $1) [20].

### Sampling

After testing, we deployed 2 pilots of IVR syndromic surveys with different levels of representativeness. The objective of the first pilot was to assess the feasibility of the IVR system to carry out syndromic surveys, as well as determine population profiles at the national level more likely to respond to this tool. The second pilot was aimed at determining whether quota sampling was effective in improving population representativeness for less populated regions. Following previous work [21-23], the 5 different regions were defined as follows: Caribbean (including Córdoba, Sucre, Bolívar, Magdalena, Atlántico, La Guajira, Cesar, and San Andrés y Providencia) with around 12 million inhabitants, Pacific region (including Chocó, Valle del Cauca, Cauca, and Nariño) with a population around 8.5 million, Amazonas river region (including Amazonas, Putumayo, Caquetá, Vaupés, Guaviare, and Guainía) with about 1.1 million population, Orinoco River region (including Meta, Vichada, Casanare, and Arauca) with 2 million population, and the Central region (including Bogotá, Cundinamarca, Huila, Tolima, Boyacá, Santander, Norte de Santander, Caldas, Risaralda, Antioquia, and Quindío) with around 29 million population [24].

The sample size for the first pilot was calculated using the STEPS (WHO Stepwise Approach to Noncommunicable Disease Risk Factor Surveillance) survey calculator as used in previous research [9,16,25,26]. The STEPS survey is a recommended calculation for repeated cross-sectional, population-based household surveys (see equation 1). The assumptions for the sample size are a 95% CI (*z* score=1.96), margin of error of 0.05, and baseline prevalence of 0.34 [27], yielding 345 complete surveys to be deployed to have nationally representative surveys.

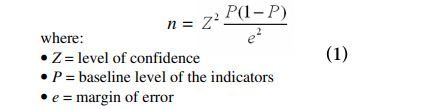



The second pilot instead focused on producing values that are representative of 5 regions in Colombia through automated strata sampling [28]. To achieve this, we recalculated the sample size with the prevalence of symptoms obtained from the previous survey (0.06), yielding a total of 87 complete surveys for each of the 5 regions, keeping all other parameters the same.

The deployment of the surveys took place between March 12, 2022, and March 15, 2022, and April 23, 2022, and May 13, 2022, respectively. The second pilot took longer because of the quota sampling, where many calls were deemed ineligible if the quota for that region had been already filled.

### Outcomes—Disposition Codes

Disposition codes were assessed using the following nomenclature. I stands for *complete interview*. The respondents were aged ≥18 years, consented, and answered all applicable questions. P stands for incomplete interview. The respondents were aged ≥18 years, consented, answered demographics questions, and some questions from the other modules, but did not finish the information on the COVID-19 module (did not reach the airtime incentive question). B stands for break-off the interview. The respondents were aged ≥18 years, consented, and answered the demographics questions. R stands for refuse after listening to the consent. U stands for unknown as these were phone numbers that did not answer or did answer but did not reach the consent and their eligibility status could not be ascertained. Respondents can be deemed ineligible for three reasons: (1) line not registered, (2) they might report being aged younger than 18 years, or (3) in pilot 2 they might be from a region whose quota sample was already met [29].

Survey rates are defined as follows: (1) contact rate measures the proportion of all cases in which some eligible mobile phone user was reached by the survey (I+P+B+R/I+P+B+R+U). (2) Response rate measures the proportion of both complete and incomplete interviews divided by all known eligible mobile phone users and users with unknown eligibility (I+P/I+P+B+R+U). (3) Refusal rate measures the proportion of mobile phone users that do not consent or break off the survey after consent divided by all known eligible users and users with unknown eligibility (B+R/I+P+B+R+U). (4) Cooperation rate: measures the proportion of complete interviews divided by all known eligible mobile phone users. (I/I+P+B)

**Figure 1:**
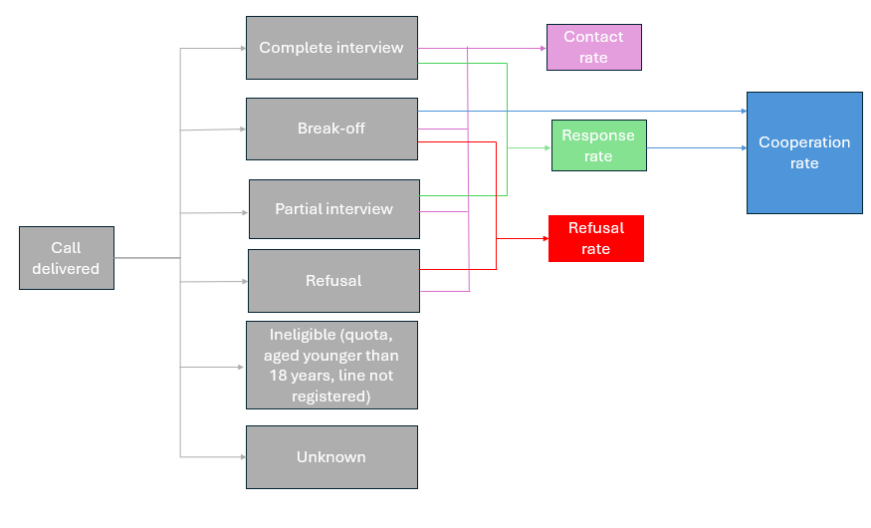
Outline for the disposition codes.

An outline for the disposition codes can be seen in Figure 1.

### Outcomes—Demographic Characteristics

Demographic characteristics of the respondents were compared to the National Quality of Life Survey 2021 (NQLS). Importantly, we did not use statistical tests to compare the 2 databases given that the sources of data are different in structure (Table 1).

**Table 1:** Comparison of sociodemographic and vaccination variables for Colombia, pilot 1^a^.

Variables			NQLS 2021^b,c,d^, % (SE)		Pilot 1 (N=367), n (%, SE)	Point difference between NQLS and pilot 1
**Sociodemographic**						
	Age (years), mean (SD)	41.8 (0.068)		36.3 (0.734)		–5.4
**Age groups (years)**						
	18-29	27 (0.0019)		139 (38, 0.025)		+11
	30-39	21 (0.0018)		88 (24, 0.022)		+3
	40-49	18 (0.0017)		70 (19, 0.020)		+1
	50-59	16 (0.0015)		37 (10, 0.015)		–6
	60-78	18 (0.0016)		33 (9, 0.014)		–9
Sex (female)			52 (0.002)		209 (57, 0.025)	+5
**Geographical area**						
	Rural	22 (0.0012)		99 (27, 0.023)		+5
	Urban	78 (0.0012)		268 (73, 0.023)		–5
**Education level**						
	Do not have	5 (00007)		26 (7, 0.012)		+2
	Elementary school	24 (0.0017)		48 (13, 0.017)		–11
	High school	44 (0.0022)		128 (35, 0.024)		–9
	Technical	12 (0.0016)		99 (27, 0.023)		+15
	Undergraduate or more	15 (0.0019)		66 (18, 0.020)		+3
**Vaccinated against COVID-19**						
	Yes	—^e^		330 (90, 0.015)		—
	No	—		37 (10, 0.015)		—

^a^We do not use tests to compare both databases given that both sources of data are different in structure and cannot be merged in the same database.

^b^NQLS: National Quality of Life Survey 2021.

^c^For the National Quality of Life Survey, absolute values are not reported because we estimated weighted percentages.

^d^The percentages of the National Quality of Life Survey 2021 were calculated with sample weights.

^e^Not applicable.

All statistical analyses were conducted using Stata (version 14; StataCorp LLC).

### Ethical Considerations

This study was approved by the Institutional Review Board of the Johns Hopkins Bloomberg School of Public Health (17868), and the Ethics Committee of the Public Health Institute at Universidad Javeriana under filing number 4 of the session conducted on August 13, 2021.

## Results

### Disposition Codes and Survey Rates for Pilots 1 and 2

#### Pilot 1

In total, 55,000 phone calls were made for the first pilot. The contact rate was 1.64% (900 out of 55,000), the response rate was 0.73% (403 out of 55,000), and the cooperation rate was 80.48% (367 of 456; see Tables 2 and 3).

**Table 2:** Disposition codes and survey rates for pilots 1 and 2^a^.

Call outcomes	Pilot 1 (N=55,000), n (%)	Pilot 2 (N=588,891), n (%)
Complete interview	367 (0.7)	451 (0.08)
Partial interview	36 (0.07)	24 (0.004)
Break-off	53 (0.10)	72 (0.01)
Ineligible: quota met	N/A^b^	3486 (0.59)
Ineligible: <18 years	72 (0.13)	633 (0.11)
Ineligible: line not registered	10,891 (19.80)	119,542 (20.30)
Refusal	444 (0.81)	6230 (1.06)
Unknown	43,137 (78.43)	458,453 (77.85)

^a^The disposition codes of the pilot surveys are according to the American Association for Public Opinion Research [29].

^b^N/A: not applicable.

**Table 3:** Disposition codes and survey rates for pilots 1 and 2^a^.

Survey rate	Pilot 1, n/N (%)	Pilot 2, n/N (%)
Contact rate^b^	900/43,984 (2.05)	6777/465,230 (1.46)
Response rate^c^	403/44,037 (0.92)	475/465,230 (0.10)
Refusal rate^d^	497/44,037 (1.13)	6302/465,230 (1.35)
Cooperation rate^e^	367/456 (80.48)	451/547 (82.45)

^a^The disposition codes of the pilot surveys are according to the American Association for Public Opinion Research [29].

^b^Contact rate: measures the proportion of all cases in which some eligible mobile phone user was reached by the survey.

^c^Response rate: measures the proportion of both complete and incomplete interviews over both all known eligible mobile phone users and users with unknown eligibility.

^d^Refusal rate: measures the proportion of mobile phone users that do not consent or break off the survey after consent over both all known eligible and users with unknown eligibility.

^e^Cooperation rate: measures the proportion of complete interviews over all known eligible mobile phone users.

We met the sample size with 367 surveys for a nationally representative sample. The average age was 36.36 years (SD 14.06 years, range 18 to 78 years); the population with the highest participation in the sample were aged 18 to 29 (n=139, 38%) years and 30 to 39 (n=88, 24%) years, followed by 40 to 49 age group (n=70, 19%). More than half were women (n=209, 57%), had not completed a college degree (n=301, 82%), and lived in an urban area (n=268, 73%).

We found that 6% (22 out of 367) of respondents reported that someone in their household had had any COVID-19–related symptoms in the past 3 days. It was primarily the respondents who had any COVID-19–related symptoms (n=11, 52%). Likewise, 44% (n=17) of the respondents reported having had a confirmed or suspected case of COVID-19 in the past. Further, 90% (n=329) of respondents reported having received at least one dose of the COVID-19 vaccine, 54% (n=177) reported being fully vaccinated but without the booster, and 27% (n=88) stated being fully vaccinated with the booster. Of the 10% (38/367) who had not been vaccinated, they cited concerns about vaccine safety (n=10, 27%). Additionally, the top 3 means by which respondents learned about the COVID-19 vaccine were *other networks or the internet* (n=180, 49%), *Facebook* (n=77, 21%), and *friends or relatives* (n=43, 12%). Finally, most respondents reported that they clearly understood the information on COVID-19 and trusted the information they received (Table 4).

**Table 4:** COVID-19 variables for pilot 1.

Variables			Values
**Symptoms related to COVID-19 (3 days ago), n (%)**			
	Yes	21 (6)	
	No	346 (94)	
**Who has COVID-19, n (%)**			
	Respondent	11 (52)	
	Household	5 (24)	
	Respondent and household	5 (24)	
Age of sick household member (years), mean (SD)			26.4 (21.74)
Sex of sick household member (female), n (%)			4 (80)
**Community symptoms, n (%)** ^a^			
	Yes	11 (65)	
	No	6 (35)	
**Have you ever had COVID-19, n (%)**			
	Yes, confirmed	69 (19)	
	Yes, suspected	92 (25)	
	No	206 (56)	
**Wanted to receive the COVID-19 vaccine, n (%)** ^b^			
	Yes	276 (84)	
	No	53 (16)	
**Would you like to receive the COVID-19 vaccine, n (%)**			
	Yes	17 (44)	
	No	21 (55)	
**Why have you not been vaccinated, n (%)**			
	Vaccines were not available	6 (16)	
	Vaccine does not work	2 (5)	
	Vaccine is not safe	10 (27)	
	I do not think I need it	5 (13)	
	Already got COVID-19	2 (5)	
	I have not been able to get the vaccine	6 (16)	
	Other reasons	5 (13)	
	Do not know or do not want to respond	2 (5)	
**Number of COVID-19 vaccine doses, n (%)**			
	One dose	61 (19)	
	Fully, not booster	177 (54)	
	Fully, plus booster	88 (27)	
**The brand of the first vaccine dose, n (%)**			
	CoronaVac	87 (26)	
	Pfizer	78 (24)	
	Johnson & Johnson	66 (20)	
	AstraZeneca	45 (14)	
	Moderna	44 (13)	
	Another vaccine	2 (1)	
	Do not know	7 (2)	
**The brand of the second vaccine dose, n (%)**			
	CoronaVac	75 (28)	
	Pfizer	76 (29)	
	Johnson & Johnson	35 (13)	
	AstraZeneca	33 (12)	
	Moderna	37 (14)	
	Do not know	9 (3)	
**The brand of the booster vaccine dose, n (%)**			
	CoronaVac	16 (18)	
	Pfizer	24 (27)	
	Johnson & Johnson	12 (14)	
	AstraZeneca	16 (18)	
	Moderna	17 (19)	
	Do not know	3 (3)	
**Vaccination record for COVID-19, n (%)**			
	Yes, I have it	331 (90)	
	I do not have it	30 (8)	
	I do not know it	3 (1)	
	I prefer not to answer	3 (1)	
**Main source of information about COVID-19 vaccination, n (%)**			
	Facebook	77 (21)	
	WhatsApp	29 (8)	
	Other networks or internet	180 (49)	
	Friends or relatives (word of mouth)	43 (12)	
	Radio	4 (1)	
	Television	26 (7)	
	Print media	3 (1)	
	I have not received any information	5 (1)	
**Your understanding about COVID-19 vaccination seems, n (%)**			
	Very clear	244 (66)	
	It is more or less clear	92 (25)	
	Little clear	24 (7)	
	Not clear	7 (2)	
**Do you trust the information you receive about COVID-19 vaccination, n (%)**			
	Always	157 (43)	
	Almost always	135 (37)	
	Almost never	55 (15)	
	Never	20 (5)	

^a^Community symptoms: do you know about someone outside your household who has had in the last month any of the following symptoms: fever, sore throat, frequent cough, feeling sick, or loss of the sense of smell?

^b^Wanted to receive the COVID-19 vaccine: did you want to receive the COVID-19 vaccine?

#### Pilot 2

In total, 588,891 phone calls were made for the second pilot. The contact rate was 1.16% (6777 out of 588,891), the response rate was 0.08% (474 out of 588,891), and the cooperation rate was 82.45% (451 out of 547; see Tables 2 and 3).

We met the sample size for the 5 regions in Colombia (Caribbean region: n=90, Pacific region: n=91, Amazon region: n=88, Orinoco River region: n=93, and Central region: n=89). In total, for the 5 regions, a sample of 451 responses was reached. All results are presented in Table 5.

**Table 5:** Comparison of sociodemographic and vaccination variables for Colombia, pilot 2.

Variables		The Caribbean region			The Pacific region			The Amazon region				The Orinoquia region				The Central region		
		NQLS^a,b,c^ 2021, % (SE)	IVR^d^ (n=90), % (SE)	Difference between NQLS and pilot 2	NQLS 2021, % (SE)	IVR (n=91), % (SE)	Difference between NQLS and pilot 2	NQLS 2021, % (SE)	IVR (n=88), % (SE)	Difference between NQLS and pilot 2	NQLS 2021, % (SE)		IVR (n=93), % (SE)	Difference between NQLS and pilot 2	NQLS 2021, % (SE)		IVR (n=89), % (SE)	Difference between NQLS and pilot 2
**Sociodemographic**																		
	Age (years), mean (SD)	42.04 (0.101)	36.16 (1.623)	–5.88	43.43 (0.167)	34.89 (1.367)	–8.41	40.41 (0.152)	29.72 (1.115)	–10.69	41.01 (0.171)		32.69 (1.286)	–8.32	43.52 (0.111)		34.17 (1.491)	–9.35
**Age groups (years)**																		
	18-29	29 (0.0028)	41 (0.052)	12	27 (0.0043)	40 (0.051)	13	32 (0.0045)	52 (0.053)	20	31 (0.0047)		48 (0.052)	17	26 (0.0029		46 (0.053)	20
	30-39	21 (0.0025)	24 (0.045)	3	21 (0.0039)	25 (0.045)	4	22 (0.0040)	28 (0.048)	6	22 (0.0042)		26 (0.045)	4	21 (0.0027)		20 (0.042)	–1
	40-49	18 (0.0024)	15 (0.037)	–3	17 (0.0036)	23 (0.044)	6	17 (0.0035)	16 (0.039)	–1	18 (0.0039)		13 (0.034)	–5	18 (0.0025)		16 (0.038)	–2
	50-59	15 (0.0021)	11 (0.033)	–4	15 (0.0033)	9 (0.029)	–6	14 (0.0031)	2 (0.015)	–12	13 (0.0034)		11 (0.032)	–2	15 (0.0023)		10 (0.032)	–5
	60-91	17 (0.0022)	9 (0.030)	–8	20 (0.0038)	3 (0.018)	–17	15 (0.0031)	1 (0.011)	–14	16 (0.0037)		2 (0.015)	–14	20 (0.0025)		8 (0.028)	–12
Sex (female)		52 (0.0030)	59 (0.053)	7	53 (0.0048)	52 (0.052)	–1	51 (0.0047)	51 (0.054)	0	50 (0.0051)		49 (0.052)	–1	53 (0.0033)		64 (0.051)	11
**Geographical area**																		
	Rural	26 (0.0023)	19 (0.041)	–7	33 (0.0037)	30 (0.048)	–3	42 (0.0045)	35 (0.051)	–7	28 (0.0037)		25 (0.044)	–3	16 (0.0014)		25 (0.045)	9
	Urban	74 (0.0023)	81 (0.041)	7	67 (0.0037)	70 (0.048)	3	58 (0.0045)	65 (0.051)	7	72 (0.0037)		75 (0.044)	3	84 (0.0014)		75 (0.045)	–9
**Education level**																		
	Do not have	9 (0.0015)	4 (0.021)	–5	5 (0.0017)	4 (0.021)	–1	7 (0.0022)	6 (0.024)	–1	5 (0.0020)		3 (0.018)	–2	3 (0.0010)		2 (0.015)	–1
	Elementary school	23 (0.0025)	8 (0.028)	–15	30 (0.0042)	8 (0.028)	–22	37 (0.0047)	10 (0.032)	–27	29 (0.0045)		12 (0.033)	–17	24 (0.0026)		14 (0.036)	–10
	High school	45 (0.0031)	31 (0.049)	–14	44 (0.0049)	45 (0.052)	1	43 (0.0048)	30 (0.048)	–13	45 (0.0052)		34 (0.049)	–11	43 (0.0034)		47 (0.053)	4
	Technical	12 (0.0021)	30 (0.048)	18	10 (0.0034)	29 (0.047)	19	7 (0.0023)	37 (0.051)	30	11 (0.0034)		39 (0.050)	28	12 (0.0024)		19 (0.053)	7
	Undergraduate or more	11 (0.0021)	27 (0.046)	16	11 (0.0034)	14 (0.036)	3	6 (0.0023)	17 (0.040)	11	10 (0.0036)		12 (0.033)	1	18 (0.0030)		18 (0.041)	0
**Vaccination for COVID-19^e^**																		
	Yes	—^f^	96 (0.021)	—	—	82 (0.040)	—	—	78 (0.044)	—	—		90 (0.030)	—	—		94 (0.024)	—
	No	—	4 (0.021)	—	—	18 (0.040)	—	—	22 (0.044)	—	—		10 (0.030)	—	—		6 (0.024)	—

^a^NQLS: National Quality of Life Survey 2021.

^b^The percentages of the National Quality of Life Survey 2021 were calculated with sample weights.

^c^For the National Quality of Life Survey, absolute values are not reported because we estimated weighted percentages.

^d^IVR: interactive voice response.

^e^Vaccination data were compared with information published in Our World in Data. There should be at least one dose of vaccination. There is no available data from government sources.

^f^Not applicable.

For the 5 regions, we found that between 6% (5/90) to 12% (11/93) of respondents reported that someone in their household had had any COVID-19–related symptoms in the past 3 days. For the 5 regions, more than 50% (374/451) of the respondents reported that they did not have confirmed or suspected COVID-19 in the past. The COVID-19 vaccination rate varied across regions, from 78% (69/88) in Amazonas to 96% (86/90) in the Caribbean. Most regions reported close to 50% (221/451) full vaccination but without the booster. Of those who had not been vaccinated, the main reason cited was about vaccine safety in all regions. Additionally, the top 3 main sources of information about COVID-19 vaccination were other networks or the internet, Facebook, and friends or relatives (336/451, 74%; Table 6).

**Table 6:** COVID-19 variables for pilot 2.

Variables			The Caribbean region (n=90)		The Pacific region (n=91)		The Amazon region (n=88)		The Orinoquia region (n=93)		The Central region (n=89)
**Symptoms related to COVID-19 (3 days ago), n (%)**											
	Yes	5 (6)		6 (7)		7 (7)		11 (12)		5 (6)	
	No	85 (94)		85 (93)		82 (93)		82 (88)		84 (94)	
**Who has COVID-19, n (%)**											
	Respondent	1 (20)		1 (17)		3 (50)		5 (46)		2 (40)	
	Household	4 (80)		5 (83)		3 (50)		3 (27)		2 (40)	
	Respondent and household	—^a^		—		—		27 (3)		20 (1)	
Age of sick household member, mean (SD)			17 (16.51)		34 (30.76)		22.33 (9.07)		32.33 (24)		17.5 (17.67)
Sex of sick household member (female)			4 (100)		3 (60)		1 (33)		2 (67)		1 (50)
**Community symptoms** ^b^ **, n (%)**											
	Yes	1 (50)		1 (33)		1 (20)		6 (75)		—	
	No	1 (50)		2 (67)		4 (80)		2 (25)		4 (100)	
**Have you ever had COVID-19, n (%)**											
	Yes, confirmed	16 (18)		17 (19)		12 (14)		12 (13)		20 (22)	
	Yes, suspected	28 (31)		25 (27)		32 (36)		27 (29)		21 (24)	
	No	46 (51)		49 (54)		44 (50)		54 (58)		48 (54)	
**Wanted to receive the COVID-19 vaccine** ^c^ **, n (%)**											
	Yes	70 (81)		60 (80)		51 (74)		68 (81)		69 (82)	
	No	16 (19)		15 (20)		18 (26)		16 (19)		15 (18)	
**Would you like to receive the COVID-19 vaccine, n (%)**											
	Yes	1 (25)		3 (19)		7 (37)		3 (33)		1 (20)	
	No	3 (75)		13 (81)		12 (63)		6 (67)		4 (80)	
**Why have you not been vaccinated, n (%)**											
	Vaccines were not available	—		12 (2)		—		22 (2)		—	
	Vaccine does not work	—		7 (1)		11 (2)		—		—	
	Vaccine is not safe	3 (75)		9 (57)		10 (53)		4 (45)		3 (60)	
	I do not think I need it	—		2 (12)		—		1 (11)		1 (20)	
	Already got covid	—		—		1 (5)		—		—	
	I have not been able to get the vaccine	—		—		5 (26)		2 (22)		—	
	Other reasons	1 (25)		2 (12)		1 (5)		—		1 (20)	
	Do not know or do not want to respond	—		—		—		—		—	
**Number of COVID-19 vaccine doses, n (%)**											
	One dose	19 (22)		15 (20)		18 (26)		15 (18)		19 (23)	
	Fully, not booster	42 (50)		49 (49)		35 (51)		50 (62)		45 (54)	
	Fully, plus booster	24 (28)		23 (31)		16 (23)		16 (20)		20 (24)	
**The brand of the first vaccine dose, n (%)**											
	CoronaVac	17 (20)		15 (20)		1 (2)		19 (23)		20 (24)	
	Pfizer	23 (27)		16 (21)		14 (20)		22 (26)		19 (23)	
	Johnson & Johnson	18 (21)		25 (33)		16 (23)		11 (13)		22 (26)	
	Astra Zeneca	14 (16)		7 (10)		16 (23)		16 (19)		7 (8)	
	Moderna	12 (14)		11 (15)		9 (13)		14 (17)		15 (18)	
	Another vaccine	1 (1)		—		11 (16)		—		—	
	Do not know	1 (1)		1 (1)		2 (3)		2 (2)		1 (1)	
**The brand of the second vaccine dose, n (%)**											
	CoronaVac	12 (18)		11 (19)		10 (20)		3 (4)		17 (26)	
	Pfizer	23 (35)		15 (25)		13 (25)		20 (30)		17 (26)	
	Johnson & Johnson	8 (12)		11 (19)		8 (16)		17 (26)		10 (15)	
	Astra Zeneca	11 (17)		9 (15)		8 (16)		2 (3)		7 (11)	
	Moderna	10 (15)		12 (20)		11 (21)		13 (20)		12 (19)	
	Do not know	2 (3)		1 (2)		1 (2)		11 (17)		2 (3)	
**The brand of the booster vaccine dose, n (%)**											
	CoronaVac	2 (8)		3 (13)		3 (19)		2 (12)		5 (1)	
	Pfizer	12 (50)		5 (22)		8 (50)		6 (38)		9 (45)	
	Johnson & Johnson	4 (18)		4 (17)		2 (12)		2 (12)		2 (10)	
	Astra Zeneca	2 (8)		1 (4)		1 (7)		3 (20)		4 (20)	
	Moderna	2 (8)		3 (13)		2 (12)		2 (12)		2 (10)	
	Do not know	2 (8)		7 (31)		—		1 (6)		1 (5)	
**Vaccination record for COVID-19, n (%)**											
	Yes, I have it	80 (89)		71 (78)		66 (75)		80 (86)		82 (92)	
	I do not have it	8 (9)		14 (16)		16 (18)		11 (12)		7 (8)	
	I do not know it	2 (2)		3 (3)		4 (5)		2 (2)		—	
	I prefer not to answer	—		3 (3)		2 (2)		—		—	
**Main source of information about COVID-19 vaccination, n (%)**											
	Facebook	16 (18)		19 (21)		18 (20)		20 (21)		17 (19)	
	WhatsApp	3 (3)		8 (9)		6 (7)		9 (10)		13 (15)	
	Other networks or internet	49 (55)		39 (43)		35 (40)		35 (38)		45 (51)	
	Friends or relatives (word of mouth)	9 (10)		8 (9)		9 (10)		10 (11)		7 (8)	
	Radio	2 (2)		5 (5)		4 (5)		3 (3)		3 (3)	
	Television	8 (9)		9 (10)		12 (14)		10 (11)		2 (2)	
	Print media	2 (2)		1 (1)		1 (1)		2 (2)		1 (1)	
	I have not received any information	1 (1)		2 (2)		3 (3)		4 (4)		1 (1)	
**Your understanding about COVID-19 vaccination seems, n (%)**											
	Very clear	63 (70)		61 (67)		52 (59)		59 (63)		58 (65)	
	It is more or less clear	19 (21)		19 (21)		24 (27)		17 (18)		23 (26)	
	Little clear	4 (4)		9 (10)		8 (9)		11 (12)		7 (8)	
	Not clear	4 (4)		2 (2)		4 (5)		6 (7)		1 (1)	
**Do you trust the information you receive about COVID-19 vaccination, n (%)**											
	Always	44 (49)		41 (45)		38 (43)		36 (39)		46 (52)	
	Almost always	30 (33)		31 (34)		26 (30)		38 (41)		18 (20)	
	Almost never	10 (11)		14 (15)		19 (22)		10 (11)		24 (27)	
	Never	6 (7)		5 (5)		5 (5)		9 (9)		1 (1)	

^a^Not applicable.

^b^Community symptoms: Do you know about someone outside your household who has had in the last month any of the following symptoms: fever, sore throat, frequent cough, feeling sick, or loss of the sense of smell?

^c^Wanted to receive the COVID-19 vaccine: Did you want to receive the COVID-19 vaccine?

### Comparison of NQLS With Mobile Phone Survey Samples

#### Pilot 1

We found that demographic variables have a similar distribution between the mobile phone surveys and NQLS. Even though respondents are more likely to be younger and female, differences do not seem to be meaningful (we are not making statements on significance because we cannot test the differences between points estimated in both surveys). Regarding geographical variables, we found that in both IVR and NQLS samples, a quarter of respondents stated living in rural areas. Importantly, IVR respondents tend to be more educated than the general population (Table 5).

#### Pilot 2

Overall, for the 5 regions, we found that demographic variables have a similar distribution to pilot 1 results. When comparing pilot 2 results to NQLS, we found that in regions with lower access to mobile phone technology, population groups are less likely to be represented in IVR surveys (older, less educated, and rural) than in regions with higher access to mobile phone technology. For example, the central region concentrates around 60% of the population of the country. In this region, the share of respondents reporting living in rural areas was 9 percentage points higher than its share of the population. Meanwhile, in the Orinoco and Amazon river regions (each with around 1.5% of the population), the share of respondents reporting living in urban areas was 3 percentage points higher than its share of the population.

## Discussion

In this study, we conducted 2 different pilots to assess the feasibility of a national active syndromic surveillance system for COVID-19 in Colombia. In both pilots, we found that it is possible to deploy a rapid active syndromic surveillance system for respiratory disease at the national level in Colombia with fair contact rates (between 1% and 2%) and excellent cooperation rates (above 80%) when compared to other studies [6]. Evidence from Burkina Faso, a low-income country, has reported similar cooperation rates using IVR [30] in a nonemergency context. In another study, an IVR survey implemented during the COVID-19 pandemic reported refusal rates in Ecuador and Sri Lanka of 5.1% and 2%, respectively, whereas our refusal rate was close to 1% (6799/509,267). Another study conducted in 5 LMICs reported similar contact, response, cooperation, and refusal rates [8].

Sampling and operational characteristics allow public health officials to monitor changes in the prevalence of respiratory symptoms over time to detect and prepare for changes in respiratory disease prevalence. An additional benefit of this technology is that it does not need face-to-face interaction reducing exposure to survey personnel, can be deployed faster in challenging or low-density environments [26], and is generally less costly [9]. However, this system has 2 main limitations. On the demand side, it is clear that respondents are more likely to be younger, female, and more educated than the average population implying that they might be overrepresented, as it has been found in other research using IVR [16,31]. To reduce selection bias, measures must be taken to improve participation from less represented groups [28]. It is possible that over time and as younger cohorts grow, issues around digital literacy and access to cell phone numbers will subside.

On the supply side, it is also clear that connectivity and mobile phone availability might play a role in survey participation [31]. To reduce selection bias associated with this effect, the second pilot included quotas by region, revealing similar patterns to those found in the first pilot (more likely to find younger, female, and more educated respondents), but still providing closer demographic estimates to those found in external surveys. The strategy of using quotas can be further expanded to smaller areas of particular interest such as municipalities, if needed.

This study is limited mainly by the availability of government data to compare results, particularly related to COVID-19 and other cases of respiratory disease, and vaccination status by region. The available information about behavior and COVID-19 in Colombia is reported on the Johns Hopkins COVID Behaviors Dashboard, which is at the national level. For example, in March 2022, the Dashboard indicated that in Colombia, about 40% of the unvaccinated wanted to get a vaccine, and our data at the national level (pilot 1) found that that was about 44% (17/38). Likewise, the Dashboard reported an ever–COVID-19 prevalence of 43%, and our data found an ever-prevalence (confirmed or suspected) of 44% (161/367), proving similar estimates with very different data sources.

This evaluation shows that using IVR as a surveillance system may be useful and feasible for conducting syndromic surveillance amid a health emergency. This study informs new areas of expansion of this work: including developing quotas for demand-side variables that affect response rates including sex, age, and educational level as well as sentinel systems so people can report on the status of their households and neighborhoods to expand the network of surveillance.

## Abbreviations

IVR: interactive voice response
LMIC: low- and middle-income country
MPS: mobile phone survey
NQLS: National Quality of Life Survey 2021
STEPS: World Health Organization Stepwise Approach to Noncommunicable Disease Risk Factor Surveillance
WHO: World Health Organization

## Data Availability

The anonymized surveillance data used in this study are available upon written request.
